# Combination of cell-penetrating peptides with nanomaterials for the potential therapeutics of central nervous system disorders: a review

**DOI:** 10.1186/s12951-021-01002-3

**Published:** 2021-08-23

**Authors:** Ying Zhang, Pan Guo, Zhe Ma, Peng Lu, Dereje Kebebe, Zhidong Liu

**Affiliations:** 1 State Key Laboratory of Component-Based Chinese Medicine, Tianjin University of Traditional Chinese Medicine, Tianjin, 301617 China; 2 Engineering Research Center of Modern Chinese Medicine Discovery and Preparation Technique, Ministry of Education, Tianjin University of Traditional Chinese Medicine, Tianjin, 301617 China; 3 School of Pharmacy, Institute of Health Sciences, Jimma University, Jimma, Ethiopia

**Keywords:** Cell penetrating peptides, Central nervous system diseases, Nanomedicine, Drug delivery

## Abstract

Although nanomedicine have greatly developed and human life span has been extended, we have witnessed the soared incidence of central nervous system (CNS) diseases including neurodegenerative diseases (Alzheimer’s disease, Parkinson’s disease), ischemic stroke, and brain tumors, which have severely damaged the quality of life and greatly increased the economic and social burdens. Moreover, partial small molecule drugs and almost all large molecule drugs (such as recombinant protein, therapeutic antibody, and nucleic acid) cannot cross the blood–brain barrier. Therefore, it is especially important to develop a drug delivery system that can effectively deliver therapeutic drugs to the central nervous system for the treatment of central nervous system diseases. Cell penetrating peptides (CPPs) provide a potential strategy for the transport of macromolecules through the blood–brain barrier. This study analyzed and summarized the progress of CPPs in CNS diseases from three aspects: CPPs, the conjugates of CPPs and drug, and CPPs modified nanoparticles to provide scientific basis for the application of CPPs for CNS diseases.

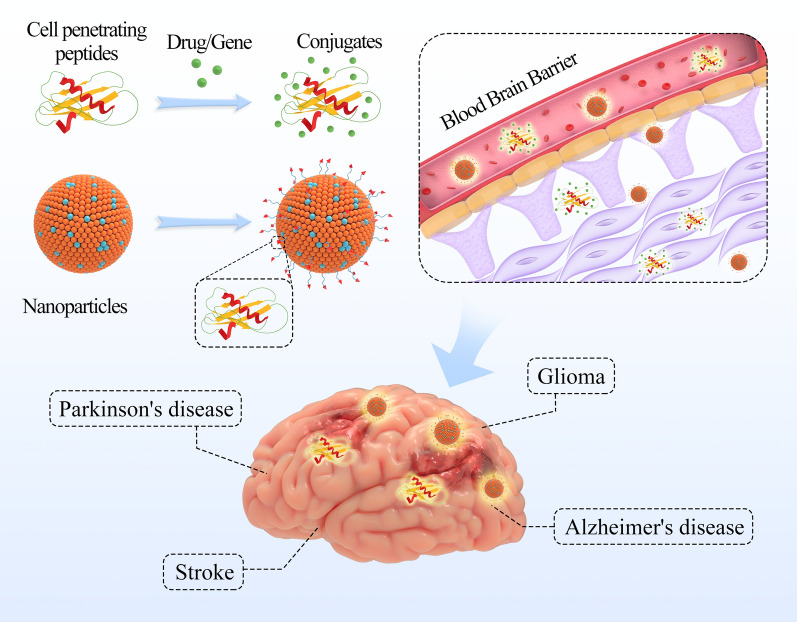

## Background

The central nervous system (CNS) is prone to a variety of structural, functional, vascular, and degenerative diseases [1, 2]. Psychiatric, neurological, developmental and substance abuse disorders affect more than 1 billion people worldwide [3]. The neurological diseases (Parkinson’s disease, Alzheimer’s disease, stroke, and brain cancers, etc.,) are the most common diseases, which seriously threatens the quality of life [4, 5]. As of 2010, CNS disorders were the leading cause of years lived with disability (YLD) globally, accounting for ~ 30% of all YLDs [6]. These dismal figures will burden the economy and damage the medical system. However, few drugs have been successfully used to treat the CNS diseases. The therapeutic effect is extremely limited by numerous factors, the biggest challenge being that drugs cannot be transported across the blood–brain barrier (BBB) [7, 8]. The transport efficiency of drugs through the BBB largely depends on the properties of size, hydrophilicity, and dissociation of the molecule. To date, most of the reported small molecule drugs and almost all large molecule drugs (recombinant protein, therapeutic antibody, nucleic acid) cannot cross the BBB [9, 10]. Therefore, the development of drug delivery systems that effectively deliver therapeutic drugs to the CNS is crucial to treat CNS diseases.

Cell-penetrating peptides (CPPs), generally composed of 5–30 amino acids, are divided into a variety of classes according to their source, structure, as well as sequence. CPPs have a strong ability to help biological materials and therapeutic drugs quickly pass-through cell membranes [11, 12], which provides a lurking strategy for transporting macromolecules through the BBB [13]. In addition, they effectively bypass the P-glycoprotein (P-gp) in the BBB [14, 15], this feature that allows them to be used as part of the delivery system for the treatment of CNS diseases. An accepted mechanism of specific CPPs is endocytosis [16]. However, the controversy of cell uptake mechanism of CPPs is remain. The CPPs are used on the basis that peptides can be attached to therapeutically active molecules and transported through cell membranes. This connection may be covalent or noncovalent [17]. In recent study, CPPs have been developed as carrier for proteins, peptides, nucleic acids, small molecule drugs, as well as nanoparticles [18]. The main limitation with CPPs as therapeutic molecular transporters is non-targeting configuration. The binding of CPPs to the targeted portion of the receptor/protein can actively deliver the molecule of interest to the desired cell at a certain concentration [19, 20].

As an emerging treatment method in medical science, nanotechnology is the manipulation of matter in the near-atomic size range to produce new structures with atomic, cellular or molecular functions. Nanomaterials have unique physical and chemical properties, such as conductivity, strength, durability and chemical reactivity, and have been used in electronic products, sunscreens, cosmetics and medicines. In recent decades, nanomaterials have attracted more and more attentions for drug /gene delivery [21, 22]. Many nanomaterials (inorganic nanoparticles [23], polymeric nanoparticles [24], micelles [25], liposomes [26], and grapheme [27], have the advantages of high drug loading, controlled drug release, good targeting, stability, biocompatibility and low toxicity [28]. In recent years, nanomaterials are proposed as a multifunctional drug delivery system through BBB, delivering loaded therapeutic drugs to CNS [29–31]. Besides, nanomaterials that avoid the uptake of reticuloendothelial system (RES) prolong the blood circulation of drug, thus significantly improving the BBB crossover opportunity of drug, leading to a high content of drugs in the brain parenchyma [32]. The advantages make nanomaterials play an important role in drug delivery through the BBB [8, 29, 32].

On the basis of nanotechnology, a lot of crossing strategies have been widely used in the transport of therapeutic drugs through the BBB [8], such as CPPs mediated BBB-crossing [33], receptor mediated BBB-crossing [34], shuttle peptide mediated BBB-crossing [35], as well as cells mediated BBB-crossing [36]. There has been an increase in the use of CPPs owing to CPPs can help drugs better penetrate blood brain barrier and enter brain lesion [37]. Drug delivery system combining CPPs with nanomaterials leads to improved performance, accuracy of drug delivery, extended half-life, stability, along with higher drug loads [8, 13]. In the article, we summarized the status of the treatment of CNS diseases, discussed the synthesis method of CPPs and the mechanism of cell uptake. Furthermore, the progress of CPPs in central nervous system diseases from three aspects: cell penetrating peptides, the conjugates of CPPs and drug/gene, and CPPs modified nanoparticles were analyzed and summarized for treatment of CNS diseases, to provide the basis for the application of CPPs in CNS diseases.

## The dilemma of treatment of CNS

### Alzheimer’s disease

Alzheimer’s disease (AD), which is closely related to the intracellular neurofibrillary tangles and the accumulation of amyloid beta in the brain, is a chronic neurodegenerative disease [38]. The effective treatment of AD is a great challenge for medical workers. At present, six kinds of drugs (such as memantine and donepezil) approved by FDA have shown certain efficacy in the clinical treatment of AD; However, they could only relieve the symptoms, but not achieve a radical cure. Two factors hinder the progress of related research. First, the etiology of AD is not fully understood by clinical medical workers. Second, the existence of blood–brain barrier limits the clinical efficacy of most drugs [39, 40].

### Parkinson disease

Parkinson's disease (PD) has a high incidence and the incidence rate is increasing year by year [41]. 1990–2015, the number of PD patients in the world increased by 118%, reaching 6.2 million [42]. PD is a common neurodegenerative disease. It is common in the elderly. The average age of onset is about 60 years old. It is rare in young people under 40 years old. The prevalence of PD is about 1.7% in people over 65 years old in China. Most patients with PD are sporadic cases, and less than 10% of them have family history. The main pathological change of PD is the degeneration and death of dopaminergic neurons in substantia nigra, which leads to the significant decrease of dopamine content in striatum. Genetic factors, environmental factors, aging and oxidative stress may be involved in the degeneration and death of dopaminergic neurons in PD [41]. In recent years, due to the poor BBB penetration of drugs, the clinical trials of drugs for the treatment of PD have been greatly hindered. Therefore, it is urgent to improve the selectivity and the BBB penetration ability of drug for PD treatment [43].

### Stroke

Stroke is mainly divided into two categories: hemorrhagic stroke (cerebral hemorrhage or subarachnoid hemorrhage) and ischemic stroke (cerebral infarction, cerebral thrombosis), of which cerebral infarction is the most common. Stroke is a cerebrovascular disease with extremely high morbidity, disability and fatality rate in the world today [44]. The middle cerebral artery is the largest branch of the internal carotid artery with a very wide range of blood supply. It is also a common site for clinical ischemic cerebrovascular disease and clinically patients with ischemic cerebrovascular disease have spontaneous reperfusion. The reperfusion of human body causes a series of secondary damages, including oxidative stress [45, 46], ion imbalance and excitotoxicity [47], damage to the BBB [48], and cell death (apoptosis or necrosis). At present, the main treatment for ischemic stroke is tissue plasminogen activator thrombolysis, but the treatment window is short (≤ 4.5 h) [49].

### Brain tumor

Brain tumors can be divided into primary brain tumors and secondary brain tumors. Secondary intracranial tumors metastasize to the brain from malignant tumors in other parts of the body, such as the lung, uterus, breast, digestive tract, liver, etc., or invade the brain from the base of the skull from malignant tumors in adjacent organs [50, 51]. Most of the cases that occur in children are primary brain cancer. As for the adults, glioblastoma multiforme (GBM) is the most common intrinsic brain tumors, accounting for about 16% of all primary brain and central nervous system tumors [52]. GBM occurs almost exclusively in the brain, but they may also occur in the brain stem, cerebellum, and spinal cord. GBM is generally considered to be a manifestation of progressive anaplasia of astrocytoma, mixed astro-oligodendrocytoma, and oligodendroglioma [53]. Studies have found that, in addition to the originally thought that GBM is only derived from glial cells, they may also come from multiple cell types with neural stem cell-like properties, which are in multiple stages of differentiation from stem cells to neurons to glial cells [54, 55]. Changes in cell phenotype not only depend on differences in cell types, but also on molecular changes in signal transduction pathways to a large extent [56]. Normally, surgery is the most effective way to treat brain tumors. The current standard treatment is to maximize tumor resection under the premise of safety and avoiding aggravating neurological dysfunction. After surgery, radiation therapy and chemotherapy with oral chemotherapy drug temozolomide are administrated simultaneous [55, 57]. In the case of glioma, however, because the boundary between healthy tissue and glioma is blurred as tumors are infiltrating and usually located in important functional areas, such as motor function and sensory areas, a complete and thorough surgical resection of GBM is exceedingly difficult. It is regrettable that even cared with the standard treatment procedure, surgery combined with radiotherapy and chemotherapy, the median survival of the patients is only 14.6 months in average and the 5-year survival rate is still < 5%.

Based on the above-mentioned dilemma in the treatment of the central nervous system, improving the delivery efficiency of the drug delivery system to the brain is essential for the treatment of CNS diseases. Common approaches for the treatment of CNS diseases include bypassing the BBB and increasing the permeability of the BBB. Methods of bypassing the BBB include intracerebroventricular [58], intracerebral [59], intrathecal [60], intranasal [61] and intratympanic [62] routes of administration, by which route the drug is delivered to the brain through the sense of smell and the trigeminal nerve [63]. Modification with antibodies or ligands of high expression on brain endothelial cells can increase the permeability of the BBB [64], and the combined application of CPP is also used to improve delivery efficiency [8].

## The synthesis and uptake mechanism of cell penetrating peptides

Peptide synthesis has developed rapidly in the past few decades. The current methods of peptide synthesis can be divided into biosynthesis and chemical synthesis. With the development of gene recombination technology, in addition to traditional natural extraction methods, commonly used enzymatic methods, and fermentation methods, gene recombination methods have also been gradually applied in peptide synthesis [65]. The peptide chemical synthesis method uses protecting groups to protect the temporarily unreactive groups in the raw amino acids to ensure that the reaction proceeds according to the design direction, and the amino acid connection extension is achieved through the condensation reaction between amino acids to obtain a peptide of a specific sequence. There are two methods for peptide chemical synthesis: liquid phase synthesis and solid phase synthesis. The main difference between the two methods comes from the use of solid phase carriers.

The first CPP, discovered by Frankel et al. in 1988, was the HIV-TAT protein responsible for virus replication, which was considered to be a powerful trans- activator of viral and cellular gene expression [66]. HIV-TAT of 86 amino acid residues is taken up by the cells as viral growth factors [67]. With the development of CPPs, researchers explored the synthesis of more effective functionally penetrating peptide sequences [68]. These synthetic peptides are developed using predictive programs, rational design strategies, and even trial and error. In most cases, solid phase synthesis is used to synthesize penetrating peptides of known sequence. So far, more than 1800 CPPs sequences were studied, and more and more CPPs have been found [69]. Except for artificially synthesized CPPs, the amino acid sequences of CPPs have obvious differences, so there is controversy about the unified classification of CPPs [70]. However, according to the physical and chemical properties, there may be a relatively more reasonable classification [71]: cationic (TAT, Penetratin, Polyarginine, P22N, DPV3, DPV6, etc.), amphipathic (MPG, Pep-1, pVEC, ARF (1–22), BPrPr (1–28), MAP, VT5, Bac7, (PPR)n, etc.) and hydrophobic CPPs. The researchers collected more than 100 kinds of CPPs widely used and noted that most CPPs (DPV3, TAT, R8) had a net positive charge, while negatively charged CPPs fall into different categories based on their properties [72]. Cationic and amphipathic CPPs accounted for 85% of the total classification, while hydrophobic peptides only accounted for 15% [73].

**Fig. 1:**
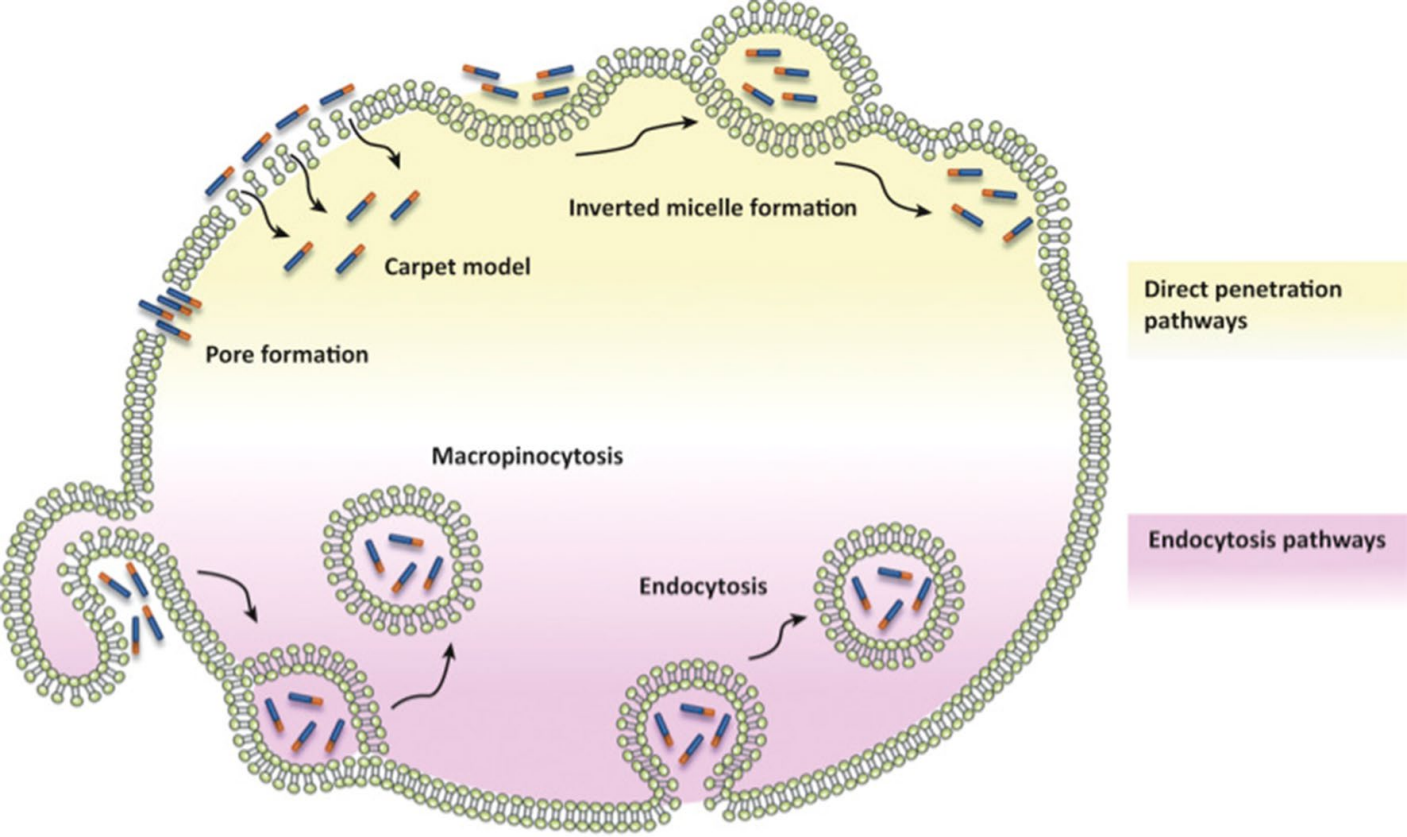
Schematic representation of proposed mechanisms for cell-penetrating peptides (CPPs) internalization. The diagram illustrates that the involved pathways can be divided into two groups: direct penetration of plasma membrane (yellow) and endocytic pathways (purple). The fifirst type of process involves several energy independent models including membrane insertion of CPPs through pore formation and membrane destabilization through the carpet-like model or inverted micelle formation. Endocytic internalization of CPPs is an energy-dependent process that comprises macropinocytosis and endocytosis. Reprinted with permission from ref. [11] Copyright (2017) Elsevier

The exact mechanism of CPPs cell infiltration remains unclear. All the time, some of differences between the studies have produced controversial conclusions. Various characteristics and conditions are critical to the efficiency of cell penetration and translocation mode, and affect different observed results, including cargo, incubation time, cell type, fluorophore, concentration of CPPs, sample handling (washing, fixation, delay), stage of cell cycle, cell density, read out (uptake distribution), etc. [74]. Currently, the different pathways can be divided into two groups [75, 76]: energy-dependent endocytosis (clathrin-mediated endocytosis, macropinocytosis, clathrin-and caveolaeindependent endocytosis and caveolae-mediated endocytosis) and energy-independent direct penetration(pore formation model, inverted micelle model and carpet model) (Fig. 1), which are mainly attributed to the characteristics of CPPs (structure, concentration, length and charge), associated cargo properties (type, charge and size) and cell types (membrane lipid composition, peptide-to-lipid ratio, and cell surface sugars) [77].

Earlier studies have shown that partial CPPs has neuroprotective effects. Fusing CPPs sequence with other peptide sequences can also improve the therapeutic effect. After forming a complex with the drug, CPPs can also enhance delivery efficiency and the concentration of drug in the brain. Furthermore, the incorporation or conjugation of CPPs with nanomaterials promotes bioavailability, stability, selectivity and in vivo efficiency [13].

## Cell penetrating peptides for CNS diseases

Peptides are compounds with certain biological activity formed by the condensation of amino acids, which have broad application prospects in the field of medicine. In some studies, peptides with penetrating and therapeutic effects can be used as drugs to treat CNS diseases.

For the treatment of GBM, one reason for the failure of the treatment is that GBM could protect themself from traditional therapies that rely on apoptotic programmed cell death by altering several signaling. Activation of mutants in the receptor tyrosine kinase (RTK) signaling pathway and phosphatidylinositol-3-OH kinase (PI3K) signaling pathway and the imbalance of tumor suppressor genes Rb and p53 play a role in promoting the pathogenesis of glioblastoma [78–80]. Like most other cancers, GBM exhibits aggressive characteristics because of its frequent dysregulation of phospholipid signaling. Due to the important role of PI3K hyperactivation in tumorigenesis and metastasis, the RTK/RAS/PI3K signaling pathway is considered to be a promising target for GBM therapy [81]. Eustace et al. reported a peptide mimetic that were derived from the phospholipid binding domain of myristoylated alanine-rich C-kinase substrate (MARCKS). MARCKS was supposed to have the potential to suppress the frequently dysregulated pathway. By exploring the selective cytotoxic effects against GBM model lines, quantifying the accumulation and localization in vitro and in vivo, and measuring its BBB, the authors explored the therapeutic potential of MARCKS peptide in the treatment of GBM. The results proved the cell permeable MARCKS peptide could effectively target all GBM molecular classes and produce rapid cytotoxicity in GBM. The MARCKS peptide showed tumor targeting properties in intracranially implanted GBM patient-derived xenograft in vivo, making it potential to be used as a GBM targeting peptide with further development [82].

Glioma initiating cells (GIC) have the characteristics of cancer stem cells and plays particularly important role in regeneration process and the resistance to radiotherapy and chemotherapy [83, 84]. However, the effective therapy fighting against GICs has not been developed. Ueda et al. synthesis a novel d-isomer peptides (dPasFHV-p53C′), which was consisted by three fragments as penetration accelerating sequence (Pas), a CPP (FHV), and C-terminus of p53 (p53C′). The synthesized peptides were effectively transduced into human GICs to explore whether it could induce cell death. The results show that dPasFHV-p53C′ would inhibit cell growth in a dose-dependent manner but have no effect on the growth of embryonic stem cells even at 3 μM when the growth of GICs was completely blocked. Interestingly, peptides with or without p53C' fragment showed the same effect, suggesting that Pas was the key factor in GIC that leads to cell death. Moreover, dPasFHV-p53C′ reduced tumor mass in GICs transplanted mice in vivo. This study demonstrates a new method for the treatment of GBM using dPasFHV-p53C' peptide transduction therapy [85].

**Fig. 2:**
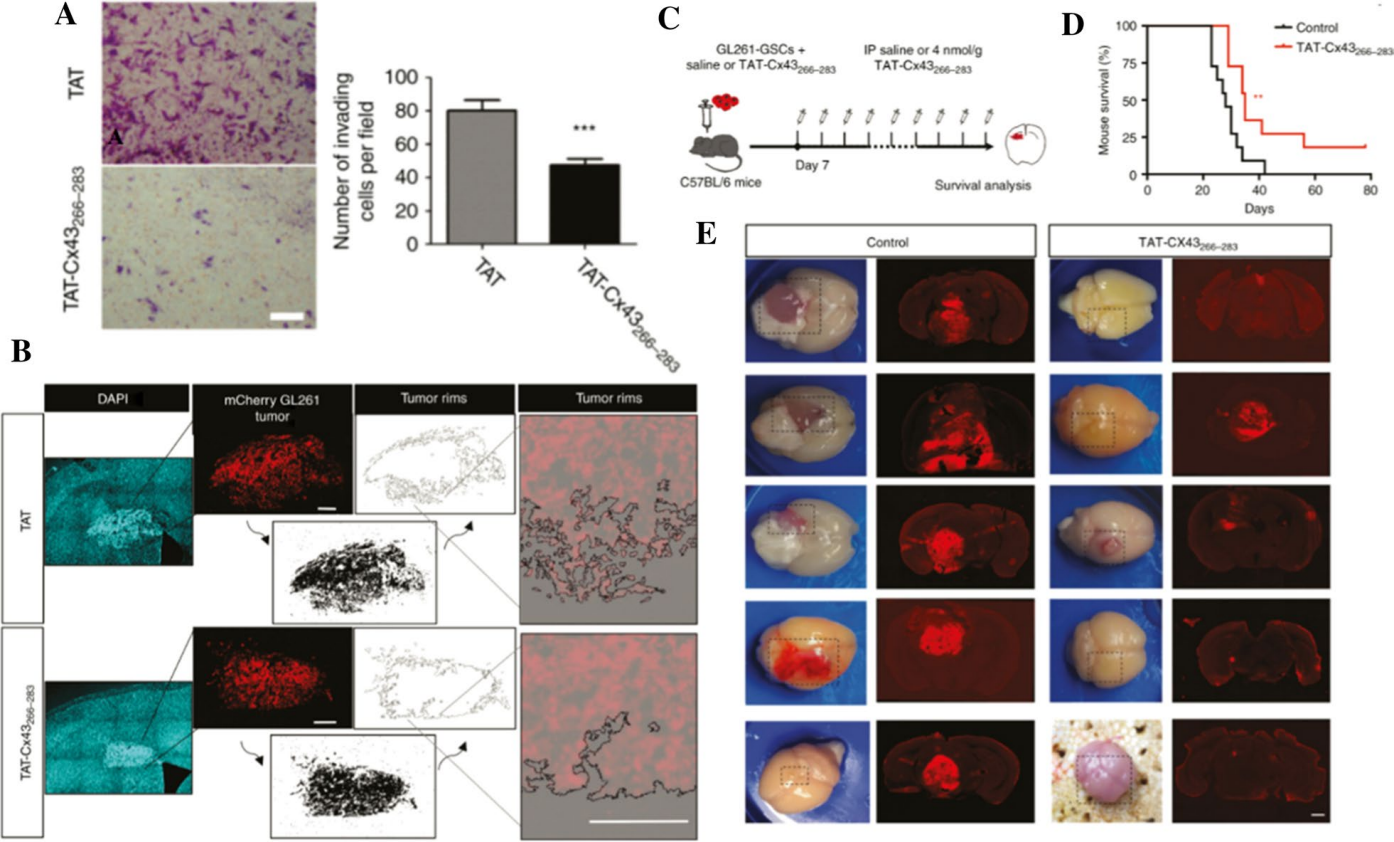
TAT-Cx43_266–283_ reduces the invasion of GL261 glioma cells in vivo. Matrigel invasion assay. GL261 cells were treated with 100 µM TAT or TAT-Cx43_266–283_ for 15 h (**A**). Mosaic immunofluorescence of DAPI (blue), mCherry GL261 glioma cells (red), the thresholded images (bottom, black), the tumor rims (top), and their magnifications (**B**). GL261-GSCs together with 100 µM TAT-Cx43_266–283_ or saline were intracranially injected in C57BL/6 mice. After 7 days, a twice per week i.p. injection of saline or 4 nmol/g TAT-Cx43_266–283_ was administered until neurological symptoms appeared (**C**). Effect of TAT-Cx43_266–283_ on the survival of mice bearing orthotopic tumor syngrafts. Percentage of animals alive along the experiment depicted in Kaplan–Meier plot (n = 11 animals per condition from 3 independent experiments). Log-rank test **P < 0.01 (**D**) Representative images of the brains and tumor-bearing brain sections from control and treated animals at the end of the experiment. Bar: 1 mm (**E**). Reprinted with permission from ref. [89] Copyright (2019) Oxford University Press Jaraíz-Rodríguez et al.

As a protein that forms gap junction channels and half channels in astrocytes, the role of connexin43 (Cx43) in malignant glioma has been deeply studied [86, 87]. Research by Jaraíz-Rodríguez et al. exhibited a kind of CPP, TAT-Cx43_266-283_, that mimics the inhibitory effect of Cx43 on c-Src inhibition [88, 89]. TAT-Cx43_266–283_ reduced the expression of nestin and Sox2 in immunodeficient mice intracranially injected with human glioma stem cells at 7 days post-implantation. TAT-Cx43266-283 also reduced the number and stemness of glioma cells 30 days after implantation and enhanced the survival of immunocompetent mice bearing gliomas derived from murine glioma stem cells. In view of the fact that TAT-Cx43266-283 could reduce the growth, invasion and development of malignant glioma, improve the survival rate of glioma bearing mice, and had no obvious toxicity to endogenous brain cells, it suggested that TAT-Cx43266-283 could be regarded as a novel clinical therapy for high-grade glioma. In further study [90], as shown in the Fig. 2 the author founds that the motility of GSCs and their invasion ability were significantly declined when incubated with TAT-Cx43266-283. The effect of TAT-Cx43266-283 on fresh excised specimens as undivided glioblastoma masses indicated that the growth, migration, and survival of these cells were significantly reduced. TAT-Cx43266-283 exhibits powerful anti-tumor effects in patient derived glioblastoma model, including GSC migration and invasion damage.

To achieve significantly inhibit the secondary inflammation and oxidative stress, and effectively reduce the extent and volume of brain edema and cerebral infarction. Zhang et al. engineered novel protein IL-1RA-PEP with potential effects of anti-inflammation and anti-oxidative stress, which fused interleukin-1 receptor antagonist (IL-1RA) with a cell penetrating peptide (PEP). Using MCAO model, IL-1RA-PEP could permeate the BBB, the cerebral infarct area in IL-1RA-PEP (50 mg/kg) group was significantly decreased compared with the vehicle group (237.5 ± 9.04 versus 334.6 ± 7.39, P < 0.001) [91]. Furthermore, Li found that, in the repeat III domain of Nuclear translocation of annexin A1(ANXA1) which participant in the neuronal apoptosis after cerebral ischemia, function as a unique nuclear translocation signal (NTS) and are required for nuclear translocation of ANXA1.The synthesized peptide Tat-NTS containing the NTS sequence of ANXA1 and the HIV-Tat cell transduction domain will specifically disrupt the interaction between ANXA1 and importin β, thereby inhibiting the nuclear translocation of ANXA1, protecting neurons from ischemic stroke deficiency. Moreover, Tat-NTS peptide has an extended efficacy with a remarkably long-time window (> 6 h) after i.c.v. administration [92].

## Cell-penetrating peptides-conjugated complex for CNS diseases

In the treatment of CNS disease, most drugs have difficulty reaching the targeted site. At this time, CPP can act as a carrier to form complexes with drugs in covalent and non-covalent forms to enhance drug delivery and achieve better therapeutic effects.

Derived from natural mammalian antimicrobial peptide protegrin-1 (PG-1), Syn-B peptide can cross the BBB through adsorption mediated endocytosis [93, 94]. By removing cysteine residues, a series of linear PG-1 analogues were synthesized and used for brain delivery. The brain's uptake of doxorubicin is significantly increased by 30 times, through the connection of l-synb1, l-synb3 and d-synb3 to doxorubicin [94]. SynB1 significantly enhanced brain uptake of benzylpenicillin without damaging the integrity of BBB [95].

Excessive accumulation of reactive oxygen species (ROS) can cause oxidative stress, which plays an important role in the occurrence and development of PD. Because mitochondria are the target of ROS damage and the site of ROS production, intracellular organelle mitochondria targeted delivery of antioxidants might prevent or reduce PD. Kang et al. [96], developed a mitochondrial targeting peptide (CAMP) with cell penetrating effect. CAMP was successfully linked to antioxidant protein human metallothionein 1a (hMT1A) and delivered to mitochondria. After incubation with CAMP-hMT1A, the production of ROS was reduced, and the mitochondrial activity and the expression of tyrosine hydroxylase were restored in PD model cells. In addition, the injection of CAMP-hMT1A into the PD mouse model could save the degeneration of dopaminergic neurons and the movement impairment.

Rusiecka et al. [97] covalently combined TP10 with dopamine to form TP10-dopamine, and experimentally verified its ability to penetrate the BBB and its anti-Parkinson activity. The results showed that in the preclinical animal model of MPTP induced PD, the amount of TP10-dopamine entering brain tissue increased, and TP10-dopamine had higher anti Parkinson’s disease activity (higher than l-DOPA). The combination of TP10 and dopamine may be a new strategy for the therapy of PD.

In another study, Vale et al. [98] linked the drug rasagiline (RAS) with penetrating peptide (MAP), named RAS-MAP, and used experiments to verify the efficacy of the newly synthesized linker. The results showed that the linkers of drugs and CPP decreased more significantly α-Syn aggregation, which may prove beneficial for PD.

Nagel linked HSP70 (the heat-shock protein 70) with Tat to form a linker Tat- Hsp70 [99]. Tat-Hsp70 protected their processes and primary mesencephalic dopaminergic neurons from MPP mediated degeneration, and transduced neuroblastoma cells in vitro. The synergistic application of HSP70 and Tat can significantly protect DA neurons in substantia nigra pars compacta against MPTP subacute toxicity in vivo. The experimental results showed that Tat-Hsp70 effectively prevents neuronal cell death.

CPPs was successfully developed to treat stroke and other brain diseases. In a study, researchers used a cell penetrating peptide (HBHAc) as a novel carrier to successfully deliver erythropoietin to the brain to demonstrate its neuroprotective effect. Compared with 24 h after occlusion, the calculated cerebral infarct area of rats treated with EPO-HBHAc was significantly reduced (29.9 ± 7.0% vs 48.9 ± 7.9%) in 3 vessels occlusion rat model [100]. In another case, researchers packaged brain-derived neurotrophic factor (BDNF) fused with CPPs (TAT and HA2) in adeno-associated virus to construct BDNF-HA2TAT/AAV to deliver BDNF intranasally to the CNS through the nasal brain route. Using chronic mild stress model, the anti-depressive behavior was observed in both tail suspension test and sucrose consumption test [100].

**Fig. 3:**
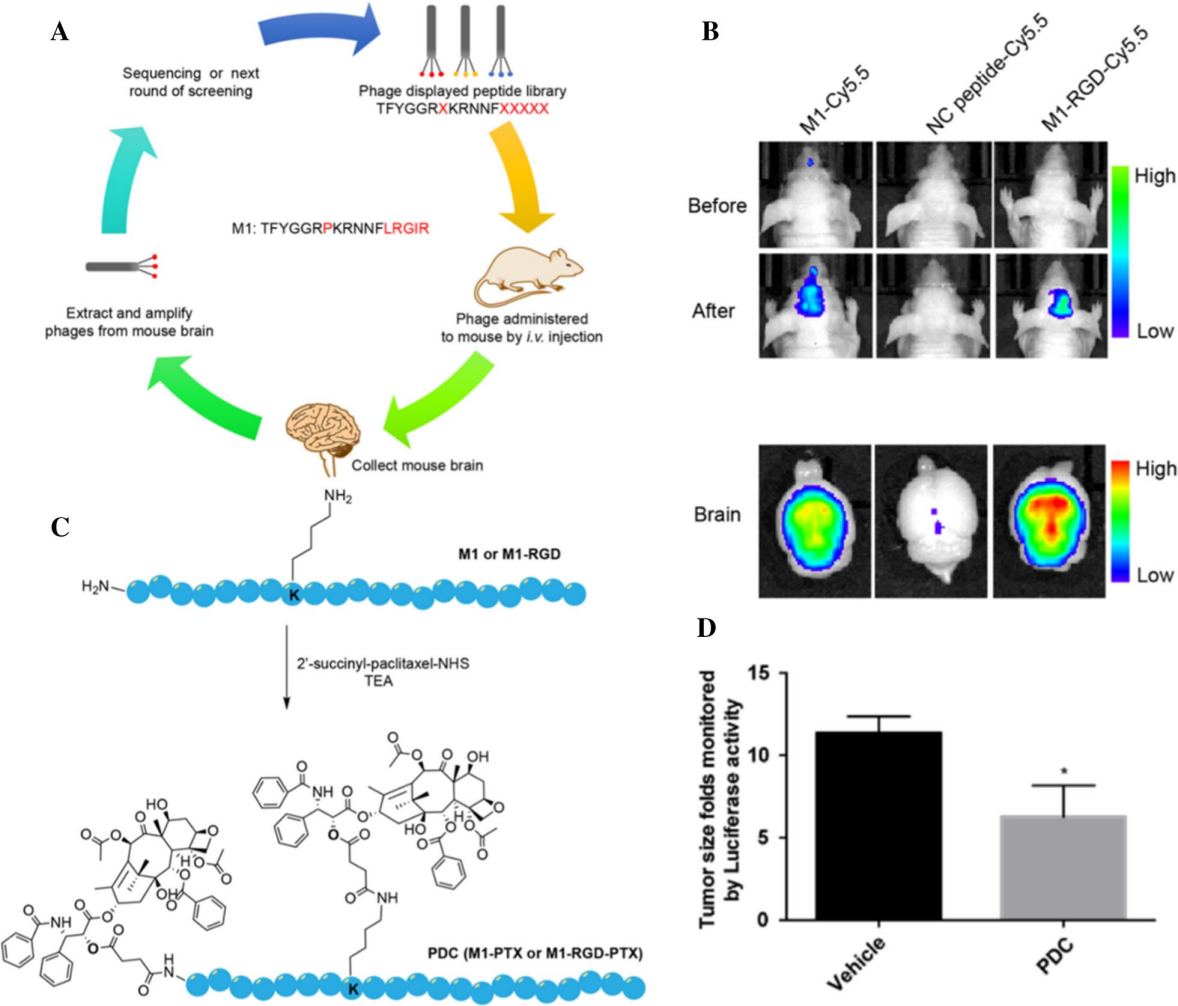
Schematic representation of the in vivo screening platform for the identifification of BBB-penetrating peptides. A novel peptide named M1 (TFYGGRPKRNNFLRGIR) was identifified and further validated (**A**). In vivo and ex vivo imaging for the validation of the BBB-penetrating ability of the peptides. A peptide with a random sequence was utilized as a negative control, and an RGD motif was appended to the C-terminus of M1 to potentially enhance its tumor targeting ability (**B**). Schematic representation of the synthesis of peptide − PTX conjugates: M1-PTX and M1-RGD-PTX. Two PTX molecules were attached to one peptide molecule (**C**). Treatment of triple negative BCBM with PDCs. Tumor suppression monitored by luciferase activity-based bioluminescence imaging (**D**). Reprinted with permission from ref. [103]. Copyright (2019) American Chemical Society

In the treatment of glioma, scientists have developed a various of effective targets based on the specific expression on the tumor surface. Low-density lipoprotein receptor-related protein-1 (LRP1) is expressed on the BBB and overexpressed in glioblastoma, which makes it the most used receptor for penetrating the BBB in drug development for the treatment of brain diseases and becomes an ideal treatment for brain cancer [101–103]. The ideal peptides that cross the BBB and bind to LRP1 were devised from a random peptide library by Chen et al. The peptide targeted and accumulated in the in-situ glioma, indicating that it could penetrate the BBB and blood–brain tumor barrier. As shown in the Fig. 3, the peptide-drug conjugate was subsequently synthesized for the treatment of glioma and breast cancer brain metastases. When combined with the clinically used chemotherapeutic drug temozolomide, it showed synergistic anti-tumor activity in both U87MG cell and MDA-MB-231BR cell models. As the peptide could shuttle compounds across the BBB, it was considered to have wide applications in the treatment of brain tumor therapies and other CNS diseases [104].

The tubulin binding sites peptide Vim-TBS.58–81, reported by Balzeau et al., was a novel CPP derived from the intermediate filament protein vimentin. It entered T98G human glioblastoma cells via endocytosis by an energy dependent process and distributed throughout the cytoplasm and nucleus and had no effect on microtubule network. When coupled to the pro-apoptogenic peptide P10 [105], the Vim-TBS.58–81 peptide localized to the nucleus and inhibits cell proliferation. These results indicated that vim-tbs.58–81 peptide was a potential transporter, which could transport peptides and possibly a wide range of cargos to the nucleus [106]. Table 1 lists more examples of CPPs used to treat CNS diseases.

**Table 1:** Cell penetrating peptides for CNS disease treatment

CPPs-cargo	CPPs	CPPs sequence	Pharmacological action	Therapeutic use	Refs.
CAMP-hMT1A	CAMP	YGRKKRRQRRRLLRAALRKAAL	Eliminate ROS and restore mitochondrial activity	PD	[158]
TP10-DA	TP10	AGYLLGKINLKALAALAKKIL	Anti-parkinsonian activity (higher than that of l-DOPA)	PD	[97]
RAS-MAP	MAP	LALKLALKALKAALKLA	Reduces α-syn aggregation	PD	[98]
SynB1-B-Pc	SynB1	RGGRLSYSRRRFSTSTGR	Increased the brain uptake of benzylpenicillin	Encephalitis	[95]
Aβ1-6A2VTAT(D)	TAT	YGRKKRRQRRR	Inhibits Aβ aggregation and cerebral amyloid deposition	AD	[159]
eGFP-TAT	TAT	YGRKKRRQRRR	Increased the amount of M receptor with modulation of acetylcholinesterase in scopolamine-induced rats	AD	[160]
JNKI	TAT	TAT (48–57)	Block the c-Jun N-terminal kinase (JNK) pathway	Cerebral ischemia	[161]
ACPP-Cy5	R9	RRRRRRRRR	Examine gelatinase activity	Image in stroke model	[162]
PTD-HA-Bcl-xL	TAT	YGRKKRRQRRR	Inhibited staurosporininduced neuronal apoptosis	Stroke	[163]
Tat-NBD	TAT	YGRKKRRQRR	Anti-NF-κB strategy	Hypoxic-ischemic (HI) brain injury	[164]
TFL457	TAT	YGRKKRRQRR	Prevents receptor disappearance from the neuronal surface	Stroke	[165]
PTD-FNK	TAT	YGRKKRRQRRR	Generate the super antiapoptotic factor	Stroke	[166]
TAT-Hsp70	TAT	RKKRRQRRR	Antiapoptotic	Cerebral ischemia	[167]
M1-RGD	M1	TFYGGRPKRNNFLRGIRSRGD	Treat glioma and triple-negative breast cancer brain metastases	Glioma	[104]
Vim-TBS.58–81-P10	Vim-TBS.58–81	Biot-GGAYVTRSSAVRLRSSVPGVRLLQ-CONH2	Inhibits cell proliferation	Glioma	[106]
gHoPe2-Dox	pVEC	LLIILRRRIRKQAHAHSK-NH_2_	Antitumor efficacy	Glioma	[168]
c[DKP-RGD]-sC18	sC18	GLRKRLRKFRNKIKEK-NH_2_	target tumor and antitumor efficacy	Glioma	[169]

## Cell-penetrating peptides modified nanoparticles for CNS diseases

The scale of nanoengineered materials enables structures to interact with biological matrices at the molecular level, providing these materials with the potential to influence changes in biological systems in unprecedented ways. Therefore, nanomaterials can be widely used in the diagnosis and treatment of CNS diseases (Table 2). In addition, nanomaterials can also be combined with other complementary technologies, such as electricity [107, 108], chemistry [109, 110], morphology [111], pulsed laser [112], near-infrared (NIR) [113] and flash photography [114], to achieve better therapeutic effects. In recent years, an increasing number of research on the combination of nanomaterials and CPPs was explored for the delivery of drugs/genes [115–119]. This combination achieves a powerful delivery effect both in vivo and in vitro. In this chapter, we reviewed combination strategies for CPPs and nanoparticles and summarized the research about CPPs modified nanoparticle in the treatment of CNS diseases based on the type of CPPs.

**Table 2:** Recent applications of nanomaterials in CNS diseases

Nanomaterial	Synthesis of nanomaterial	Cargo	Modification	Pharmacological action	Therapeutic use		Refs.
Carbon dots	Solvothermal method	–	Nitrogen (N)- containing polyaromatic	Modulating Cu(II)-mediated β-amyloid aggregation	AD		[170]
PLGA	Solvent evaporation method	Doxorubicin/lapatinib	ACUPA and cyclic TT1	Synergistic anti-tumor	Breast cancer brain metastases	[171]	
Carboxylic acid functionalized carbon dots	Acidic oxidation method	Epirubicin and temozolomide	Transferrin	Synergistic anti-tumor	Glioblastoma		[172]
TPGS-transfersomes	Thin-film hydration method	Docetaxel	Folate	Inhibits cell division and proliferation	Glioblastoma		[173]
Lipid-coating mesoporous silica nanoparticle	Stobber method and film hydration method	Paclitaxel	Angiopep-2	Inhibits cell division and proliferation	Glioma		[174]
Polyamidoamine dendrimer	Commercially available	Heme oxygenase-1 (HO-1) gene	Histidine and arginine	Anti-inflammatory and anti-apoptotic	Ischemic stroke		[175]
Graphene oxide	Modifified Hummers’ method	–	–	Inhibits amyloid beta fifibrillation	Neurodegenerative diseases		[176]
Mesoporous silica nanoparticle	Templating method	Curcumin and chrysin	–	Antioxidant	Neurodegenerative diseases		[177]
Black phosphorus	Liquid phase exfoliation method	Paeoniflorin	Lactoferrin	Attenuate deficits in tyrosine hydroxylase-positive (TH +) neuronal	PD		[178]
Selenium nanoparticles	Chemical reduction method	–	OX26	Regulate cellular metabolic state	Stroke		[179]
Nanostructured lipid carriers	Emulsifification and solvent evaporation method	Baicalin and Salvianolic acid B	OX26	Oxygen–glucose deprivation	Stroke		[180]
Fe_3_O_4_	Solvothermal method	–	PEG	Ameliorate local redox state and facilitate blood − brain-barrier recovery	Stroke		[181]

### The combination strategies for CPPs loading on nanoparticle

**Fig. 4:**
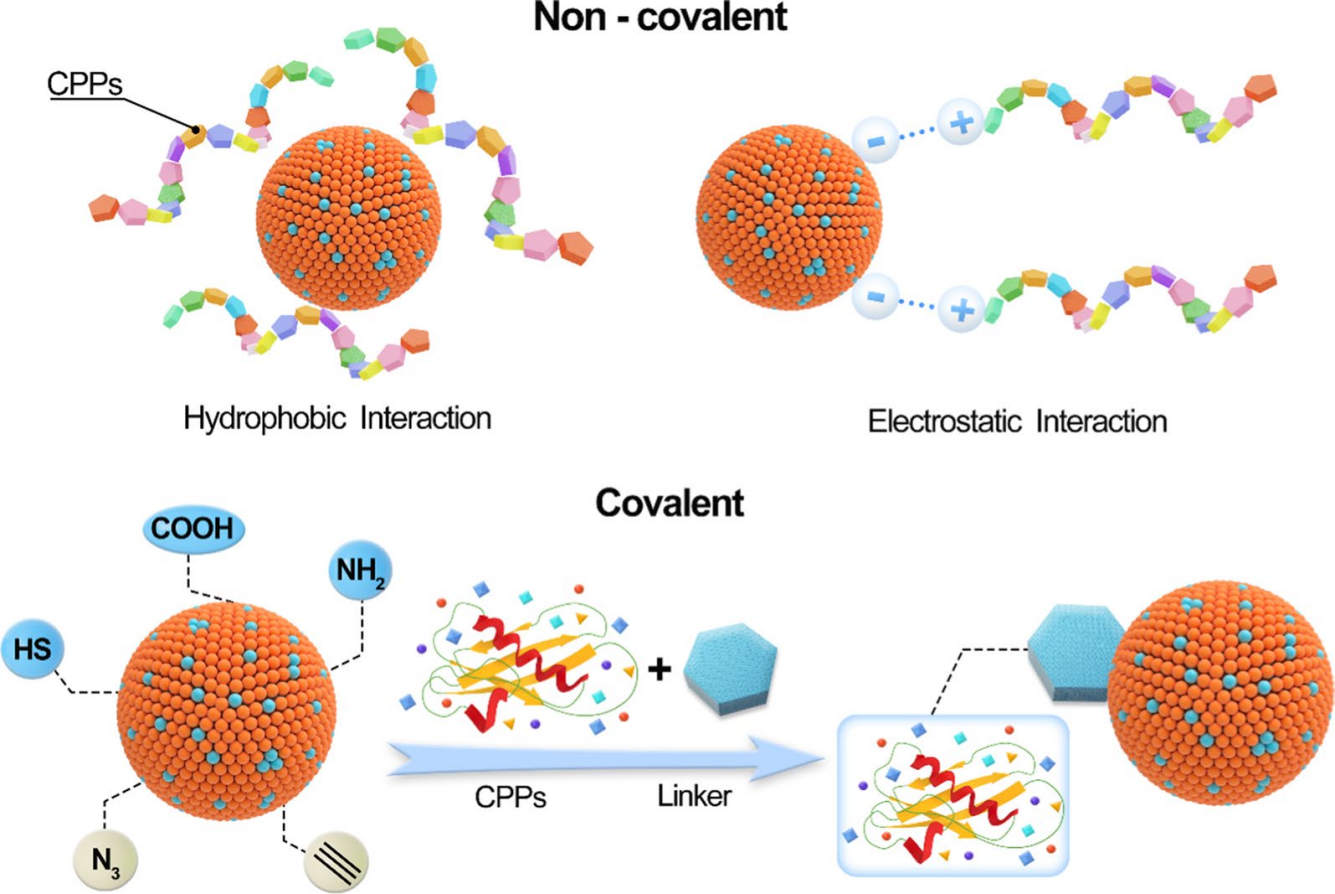
Schematic diagram of non-covalent or covalent connection between nanomaterials and CPPs

The connection of the CPPs to the nanoparticle intended for delivery into the intracellular compartment may be covalent (cleavable or non-cleavable) or may be based on non-covalent interactions (Fig. 4). The reactive group (mainly Amino, sulfhydryl, carboxyl) on CPPs provides the possibility for covalent coupling. Nanoparticles can be covalently coupled to CPPs through chemical bonds (mainly disulfide bonds or thioester bonds) or through the cloning and subsequent expression of CPPs fusion proteins. However, a major risk of covalent CPPs technology is that, in some cases, it may change the biological activity of the conjugate [11]. Linkage is usually used to adjust the optimal distance between the CPPs and the nanoparticle. This linkage can be attached to CPPs side chain functional groups, such as lysine amino or cysteine thiol groups, or even carboxyl or amino groups at the C or N-terminus of the peptide.

**Fig. 5:**
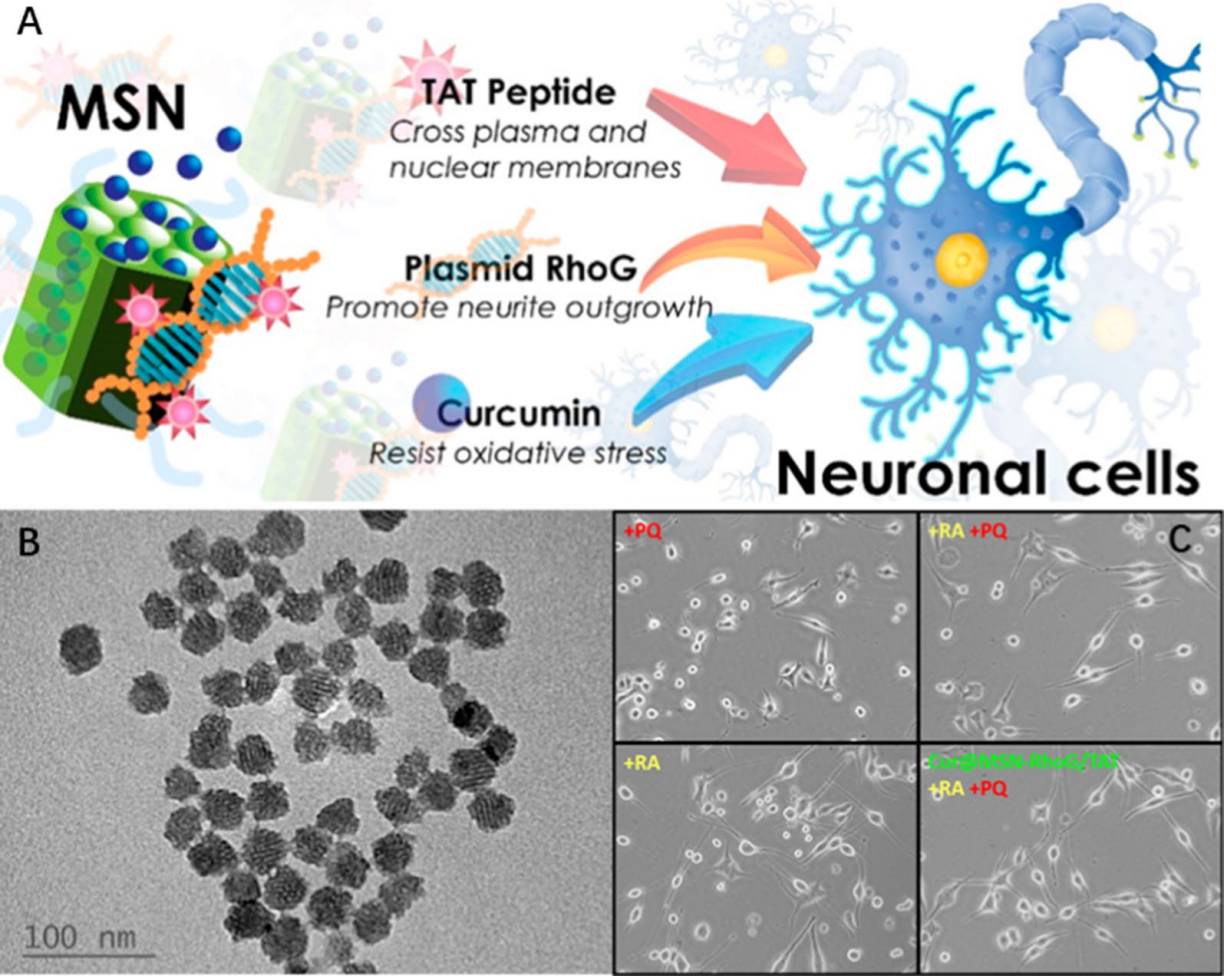
A mesoporous silica nanoparticle (MSN)-based approach (Cur@MSN-RhoG/TAT) for neurodegenerative therapy. Non-covalent binding of nanoparticles, cell penetrating peptides (TAT), gene(Plasmid RhoG) and drug(Curcumin) (**A**). TEM image of MSN (**B**). Therapeutic effffect of Cur@MSN-RhoG/TAT on neurite growth. Morphological images after 4 days of treatment with and without PQ (paraquat, generator of superoxide anion radicals, 150 μM), RA (Retinoic acid, promote neuronal difffferentiation and neurite outgrowth, 20 μM), and Cur@MSN-RhoG/TAT (256 μg/mL). Reprinted with permission from ref. [119] Copyright (2019) American Chemical Society

Compared with covalent bonding, the main advantage of non-covalent bonding is that the complex between CPP and cargo is only formed when the two components are mixed. In a study, positively charged CPP (TAT) and negatively charged plasmids are connected by electrostatic interaction (Fig. 5), and then incubated with mesoporous silicon nanoparticles with positive charge to complete the modification of the nanoparticles [120]. The prepared nanoparticles have a significant therapeutic effect on neurite growth. However, the disadvantage of non-covalent linkage is the low stability of the complex in the body environment.

### Modification of different cell penetrating peptides

#### Arginine oligomer

As short cationic peptides capable of traversing the plasma membranes, arginine-rich cell-penetrating peptides are very promising tools for the delivery of therapeutic macromolecules such as peptides, proteins, and nucleic acids [121, 122]. These peptides could effectively transport cargo through the membrane by the endocytic pathway.

Yuan et al. constructed an octaarginine R8-conjugated oleic acid-modified liposome (R8PLP) for delivering doxorubicin, which was formed from 1,2-dioleoyl-3-trimethylammonium-propane chloride, PEG-DSPE, cholesterol and phosphatidylcholine [123]. Compared with unmodified liposomes, the uptake of R8PLP by U87-MG cells increased by 8.6 times, and the cell viability was reduced by 16.18% after 24 h treatment at 3.6 μM. The AUC_0.5–12 h_ value of doxorubicin-loaded R8PLP preparation was 2.4 times higher than that of unmodified liposomes, and the biodistribution in the brain was significantly improved. Zhang et al. employ R8 to deliver microRNA (miRNA) into the brain for treating glioma [124]. MiRNA, a kind of short noncoding RNAs, can regulate gene expression of multiple target genes after transcriptional regulation, block mRNA translation and lead to mRNA degradation. MiRNA plays a causative role in the development of cancer [125]. Anti-miRNA s are single stranded nucleic acid sequences, which can prevent miRNA from recognizing the mRNA target, thus blocking the elevated miRNAs without causing mRNA degradation [126]. MiR-21 is highly expressed in glioblastoma cells, which could promote glioma invasiveness by targeting matrix metalloproteinase (MMP) [127]. To lower the miR-21 level and inhibit the development of glioblastoma, the anti-miR-21 was coupled with R8 to form complexes, which could be administered by intracranial infusion to brain tumor patients. The anti-miR-21/R8 preparation elicited efficient downstream gene upregulation, and the glioblastoma cell migration was inhibited by 25%. The results indicated that R8, as a cell penetrating peptide, can effectively promote the brain delivery of miRNA.

To enhance the tumor targeting efficiency, Wang et al. employed R8 and transferrin (Tf) for delivering doxorubicin to glioma [128]. The prepared DOX-Tf-LPs exhibited a sustained-release profile and a good uptake rate by U87 and GL261 cells. What is more, the DOX-Tf-LPs showed excellent anti-glioma efficacy while indicated low systemic toxicity by measuring the histology of major organs and the bodyweight of mice. In another case, Sharma et al. evaluated potential application of bi-ligand liposomes (R8-Tf-LPs), constituted by transferrin and poly-l-arginine co-modified liposomes, for delivering β-gal plasmid (pDNA) to brain [129]. In vivo biodistribution experiments have shown that compared with single ligands (Tf-LPs) or unmodified liposomes, R8-Tf-LPs accumulated in rat brain at significantly higher concentrations and cause the increasing expression of β-galactosidase plasmid in rat brain tissue. Hemolysis assay and histological examination authenticated the biocompatibility of bi-ligand liposomes. Co-modification of Tf and C8 enables liposomes to enhance their transfection potential and penetration ability to brain. Tian et al. introduced a multifunctional polymeric micelle coupling with arginine-glycine repeats (RG)5 and histidine-glutamic acid repeats (HE)5 [130]. (RG)5, hired as cell-penetrating peptide, and (HE)5, used as a pH-sensitive masking sequence, were incorporated into the micelles. The multifunctional polymer micelles improved cellular uptake through the effect of (RG)5, and introduced charge shielding under the influence of (HE)5 to minimize non-specific binding and uptaking under physiological pH. Through this strategy, the mixed micelles effectively conceal the cationic charges of (RG)5 at physiological pH, and showed the selective triggering characteristics in response to the weakly acidic environment in vitro based on the charge reversal mechanism. In both xenograft and orthotropic glioma mouse models in vivo, the (RG)5- and (HE)5-modified mixed micelles accumulated in tumor tissues at higher concentrations, exerting an inhibitory effect on tumor growth, but have no obvious toxicity to peripheral tissues.

As a specific ligand of integrin αvβ3 family, RGD peptides are widely used for targeting angiogenic endothelial cells and most malignant tumor cells, such as glioma cells, melanoma cells and ovarian cancer cells [131, 132]. The cyclic RGD peptide shows 1000 times of binding affinity than linear RGD peptide and exhibits advantages in glioma targeted drug delivery systems [133, 134]. Liu et al. conjugated a specific ligand cyclic RGD peptide to R8 to develop a multifunctional peptide R8-RGD [135]. The cellular uptake of R8-RGD liposomes was 2 times and nearly 30 times higher than that of separate R8 or RGD modified liposomes, respectively. In vitro cell experiments have shown that R8-RGD liposomes achieved efficient penetration in both BBB models and three-dimensional glioma spheres. While in vivo cell experiments laid out further proofs that liposomes could also selectively accumulate in glioma foci of C6 glioma bearing mice after systemic administration. When paclitaxel (PTX) was encapsulated, R8-RGD liposomes could induce the strongest inhibitory and apoptotic effects on C6 cells and prolong the survival time of mice with intracranial C6 glioma. The research by Qiu et al. and Liu et al. showed a possibility of an RGD reverse sequence dGR in use of developing active-targeting liposome R8dGR-Lip [136, 137]. The dGR was conjugated to R8 to form a tandem peptide R8-dGR, which could bind to both integrin αvβ3 and NRP-1 receptors. R8-dGR-Lip shows high penetration ability in vitro and treatment efficiency for glioma models in vivo. PTX-R8-dGR-Lip, the paclitaxel loaded liposomes, increased cellular uptake and effective penetration into glioma spheres, and induced the strongest tumor inhibition effects by anti-tumor cells, anti-tumor stem cells, as well as anti-vasculogenic mimicry in vitro. In vivo examines further explored the inhibition efficiency on C6 glioma recurrence models, illustrating that PTX-R8dGR-Lip could significantly inhibit tumor recurrence, reduce tumor tissue invasion, and prolong the survival rate of tumor-bearing mice. All the results indicated that the co-modified of R8-RGD and R8-dGR made it to become a promising solution for the BBB transporting, glioma targeting and tumor penetrating. The above studies laid foundations for further research on octaarginine as effective delivery vehicles for active compounds and genes.

#### TAT

TAT, also an arginine-rich peptide, is a basic peptide derived from human immunodeficiency virus (HIV)-1, which can transport foreign proteins into cells through cell membrane, including the BBB. TAT consists of an eight amino acids basic region with six arginine and two lysine residues, which seem to be the key to its efficient membrane transport and brain transport. Qin et al. synthesized four selected peptides [138], including a TAT peptide with terminal Cysteine, a TAT peptide with disordered sequence, a Glycine and glutamic acid substituted TAT peptide, and R8. After being covalently bonded with liposome separately, the brain targeting potential of the four peptides in vitro and in vivo were compared by investigating their ability of targeting to the brain, evaluating the cellular uptake of the four liposomes, exploring the mechanism of the pathway of endocytosis, and investigating the biodistribution in vivo. The result indicated that the charge of peptide might be a key point in enhancing its brain transmission. This sequence had little relationship with its membrane translocation, and the brain transmission suggested that TAT peptides might not have specific receptors or transporters. Also, by Qin et al., another cell-penetrating peptide TAT was covalently conjugated on the surface of liposomes encapsulated with doxorubicin (DOX) for treating brain glioma [139]. TAT modified doxorubicin liposomes showed strong anti-C6 glioma cell proliferation activity. At the same time, TAT modified liposomes could prolong the survival time of glioma bearing rats. The results of tissue distribution in brain and heart suggest that liposomes have higher brain transport efficiency and lower risk of cardiotoxicity.

As most CPPs were lack of cell selectivity and targeting effects, it is necessary to cooperate with other brain targeting ligands to achieve accurate and effective brain delivery. There are many different receptors in brain capillary endothelial cells, which are good targeting strategies. Receptor mediated cell transport (RMT) has been widely used in brain targeting research [140, 141]. Under normal conditions, specific ligands only show high affinity for targeted receptors, while are not able to enhance endocytosis or solid tumor infiltration. Combined with cell penetrating peptide, it can increase the ability of penetrating BBB and enhance the targeting. Zheng et al. designed dual-functioned liposome (Tf/TAT-lip), modified with transferrin and cell-penetrating peptide and encapsulated with doxorubicin as a model drug, for glioma chemotherapy [142]. Zheng et al. evaluated the trans-endothelial ability crossing the BBB and the anti-proliferative activity against U87 cells in vitro, and subsequently investigated the biodistribution and the anti-glioma effect by orthotropic glioma model in vivo. The positive result proves that Tf/TAT-lip could effectively deliver drugs to the brain and is expected to become a brain drug delivery system. In the study by Zong et al., a novel dual-targeting liposomal system Tf/T7-lip was prepared by conjugating transferrin T7 and TAT with liposomes [142]. The dual-targeting effects and the therapeutic effect on glioma were proved by in vivo and in vitro experiments. In three-dimensional tumor spheroid penetration studies, Tf-T7-lip could penetrate from the surface of the tumor to the core and effectively inhibit the growth of the tumor sphere. The dual-ligand liposomes demonstrated the ability to target the brain and achieve efficient delivery of tumor cells in vitro and in vivo. Compared to doxorubicin solutions and the single-ligand liposomes (Tf-lip and T7-lip), the median survival time of tumor-bearing mice treated with dual-targeting liposomes was the longest among all groups.

In addition to traditional chemotherapy, gene therapy for glioma has made some progress in related clinical research, which called for efficient gene delivery systems for glioma therapy. Gupta et al. prepared trans-activating TAT (TATp)-modified liposomes loading by plasmid encoding for the green fluorescent protein (pEGFP-N1) as the model gene and investigated the potential use to enhance the delivery in vitro and in vivo [143]. In vivo transfection of intracranial brain tumors by intratumoral injection of TATp lipid complexes showed that pEGFP-N1 was selectively delivered to tumor cells and then effectively transfected compared with ordinary plasmid lipid complexes. No transfection was observed in normal brain tissue adjacent to the tumor, indicating a better targeting efficiency to brain tumor cells. As for tumor angiogenesis therapy, Lu et al. proposed a gene therapy scheme [19]. They developed a polyethyleneimine (PEI) nanocomplex, modified by TAT and AT7, which was designed to target VEGFR-2 and neuropilin-1. The angiogenesis-inhibiting secretory endostatin gene (pVAXI-En) was loaded by the nanocomplex and then the delivery capability to glioma was evaluated through anti-angiogenic behaviors and anti-glioma effects. In both in vitro and in vivo experiments, TAT-AT7 nanocomplex could penetrate the BBB and achieve efficient gene delivery in glioma tissue, indicating its feasibility for gene therapy of glioma. Another siRNA delivery case was shown by Han et al. [144]. They constructed a novel gene delivery system, which was constituted by TAT and polyamideamide (PAMAM) dendrimers connecting with bacterial magnetic nanoparticles (BMPs). TAT-BMPs-PAMAM was assembled with small interfering RNA expression plasmid (psiRNA), and the vehicle could deliver gene to brain tumors, inhibit the expression of EGFR, down regulate the expression of tumor related proteins, and increase the number of apoptotic cells. As an efficient and targeted gene delivery system, TAT-BMPs-PAMAM showed potential of application in targeted gene therapy of brain tumors.

Apart from the method of enhancing the penetrating across the BBB, the olfactory and trigeminal nerves transport routes can deliver cargo into the brain bypassing the BBB as well. In nose-to-brain delivery system, the drug takes effect quickly after nasal administration and can enter the brain within a few minutes. Meanwhile, the first pass effect of oral administration and other gastrointestinal elimination mechanisms could also be avoided, to minimize the systemic side effects [144, 145]. Taki et al. and Kanazawa et al. focus on nose-to-brain delivery system, and designed several TAT-modified MPEG-PCL amphiphilic block copolymers as vehicles [146] to carry camptothecin (CPT), siRNA, bombesin, siRaf-1/CPT, et al. [147–151]. Taken overall, compared to intravenous administration, the amounts of cargo in the brain were significantly increased after nasal administration. In the cell uptake experiment, the uptake of TAT-modified MPEG-PCL on glioma cells was higher than that of unmodified micelles. The distribution experiment further proved that compared to MPEG-PCL, the distribution of TAT-modified MPEG-PCL was significantly higher in the brain but lower in non-target tissues, which was considered to reduce side effects and toxicity to other parts such as heart, liver, spleen, lung and kidney. When used to deliver siRNA or active ingredients, the TAT-modified MPEG-PCL micelles still exhibited remarkable capabilities in targeting delivery to brain and glioma cell penetration. The cargo, including siRNA and active ingredients, were accumulated in brain tumor tissue and achieved a good therapeutic effect on the rat model of GBM. The nose-to-brain pathway was also studied by detecting the fluorescence after intranasal administration [148] The results suggested TAT-modified MPEG-PCL could increase the efficiency of delivery from the nose to the brain by enhancing transport along the olfactory and trigeminal nerve pathways.

The above studies introduced an effective method of nose-to-brain delivery of drugs or siRNA using TAT-modified MPEG-PCL micelles. It is expected to become a new method for the treatment of brain tumors and other CNS diseases.

#### tLyp-1

Neuropilin (NRP), a transmembrane glycoprotein that traffics between cell membrane and nucleus and a member of the class 3 semaphorin family, acts as receptors for several extracellular ligands [152]. Recent studies have shown that NRP is expressed in endothelial cells and tumor cells and can be used as a receptor for various forms and isotypes of VEGF related to angiogenesis [153]. Given that NRP is highly overexpressed on the surface of glioma cells and endothelial cells of neovascularization, NRP may become a promising target for antiglioma drug delivery. It is reported that the tLyp-1 peptide, a tumor cell homing and penetrating peptide, was able to mediate tissue penetration through the NRP-1-dependent internalization pathway due to the structure of both a tumor-homing motif and a cryptic CendR motif ((R/K)XX(R/K)), which is responsible for cell internalization and tissue penetration. Hu et al. conjugated tLyp-1 peptide with PEG-PLA to form tumor homing nanoparticles for delivering drug into the glioma parenchyma [152]. After functionalized by tLyp-1 peptide, the nanoparticles enhanced the uptake of both HUVECs and rat C6 glioma cells, increased the cytotoxicity, and improved penetration and growth inhibition in avascular C6 glioma spheroids in vitro, while compared to unmodified nanoparticles. The selective aggregation and deep penetration of tLyp-1-NP at the glioma site was further confirmed by in vivo imaging and glioma distribution experiments. The intracranial C6 glioma bearing mice treated with PTX-tLyp-1-NP achieved the longest survival times. In the following study, to realize glioma cell and neovasculature dual-targeting and efficient cellular internalization, Hu et al. added a nucleolin bounding peptide F3 into drug delivery system [152, 154]. F3 peptide was discovered by using phage-displayed cDNA libraries. It was designed to specifically bind to cell surface nucleolin and can effectively realize the transport from the cell surface to the nucleus. F3-functionalized nanoparticles(F3-NP) enhanced the cellular interaction with C6 cells, and increased the cytotoxicity after loaded paclitaxel. After co-administration with tLyp-1 peptide, F3-NP showed enhanced accumulation at the tumor site, and penetrated deeply into the glioma parenchyma, and prolonged survival time in C6 glioma bearing mice. The dual strategy of simultaneously targeting glioma cells and neovasculature can significantly improve the delivery of anti-glioma drugs. In another drug delivery system modified by tLyp-1 peptide, Seleci et al. aimed to discuss the synergistic effects of combinated therapeutic agents in nanoparticles for specific glioma therapy [155]. Two active ingredient doxorubicin and curcumin were encapsulated in polyethylene glycolated niosomes (PEGNIO). Subsequently, further surface modifications were realized by use of targeting ligands tLyp-1 peptide. The in vitro cytotoxicity and growth inhibition of tumor like spheres on U87 cells and human mesenchymal stem cells clearly indicated that co-administration by doxorubicin and curcumin with tLyp-1 functionalized niosomes provided an effective strategy for the treatment of glioma.

#### CB5005

**Fig. 6:**
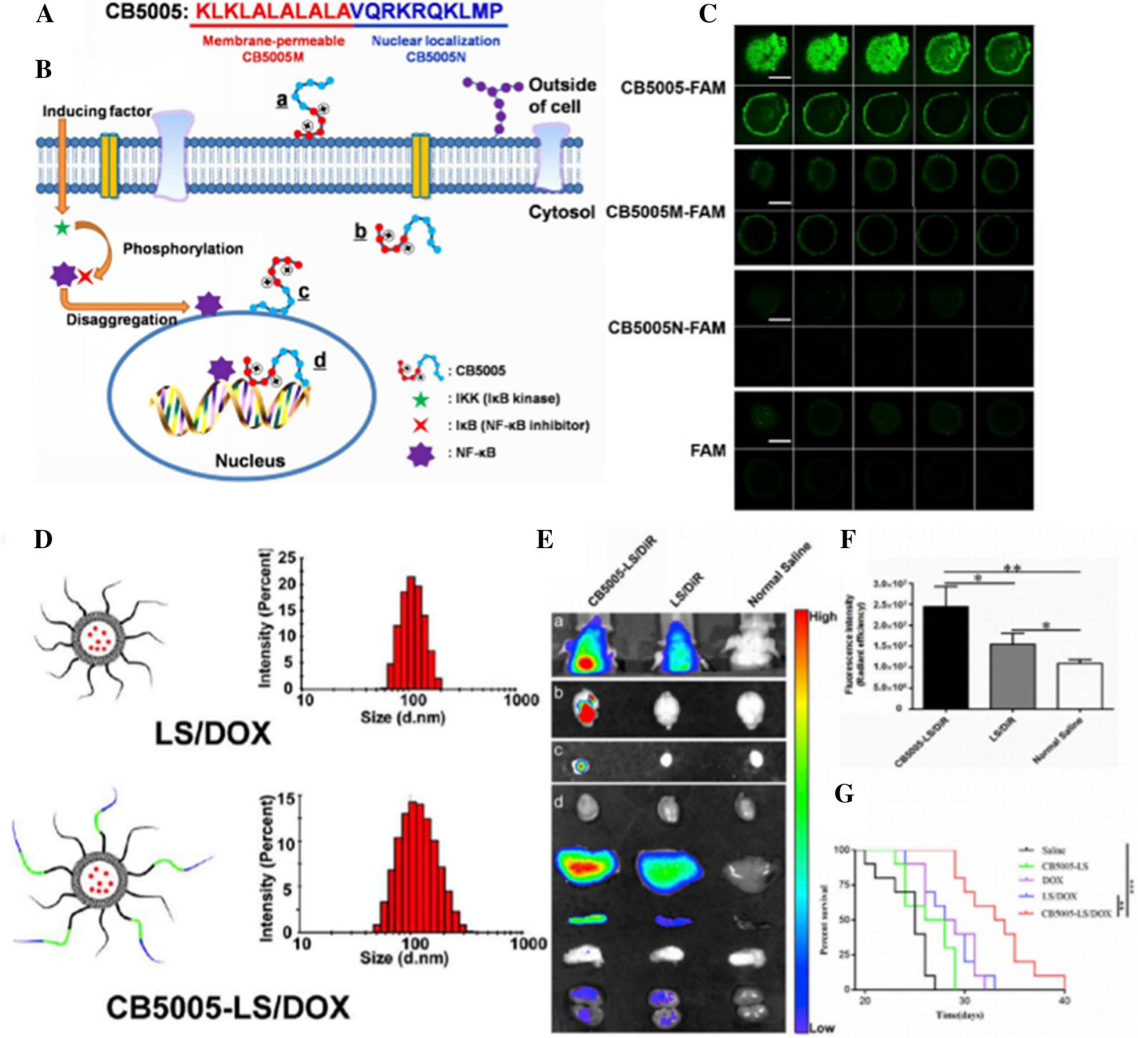
Schematic diagram showing the amino acid sequence of CB5005 (**A**), the NF-κB pathway and the approach for CB5005 to penetrate cell membrane and translocate into cell nucleus (**B**). Permeability analyses on U87 tumor spheroids after incubation for 4 h with CB5005-FAM, CB5005M-FAM, CB5005N-FAM and FAM under a concentration of 5 μM. Fluorescence images were multi-level scans of the tumor spheroids, and the interval between consecutive slides was 5 μm (**C**). Reprinted with permission from ref. [155] Copyright (2016) Elsevier. Size distribution of LS/DOX and CB5005-LS/DOX (**D**). In vivo distribution and antitumor effects of liposomes in intracranial glioblastoma-bearing nude mice. **E** The in vivo distribution of CB5005-LS/DiR and LS/DiR in intracranial glioblastoma-bearing nude mice (a) and the ex vivo distribution in brains (b), intracranial glioblastoma (c) and other organs (top to bottom were heart, liver, spleen, lung and kidney) (d). From left to right, the mice were treated with CB5005-LS/DiR, LS/DiR and normal saline, respectively. The formulations were injected via caudal vein at a dose of 100 μL with a concentration of 0.01 μM DiR. Dissected tissues, brain and intracranial glioblastoma were immediately observed at 4 h post injection. Fluorescence intensity in intracranial glioblastoma. Data were presented as mean ± SD (n = 3) (**F**). Kaplan–Meier survival curves of nude mice bearing intracranial glioblastoma (**G**). (Asterisk) Denotes statistical significance, **p < .01, and ***p < .001. Reprinted with permission from ref. [156]. Copyright (2018) Elsevier

The lack of tissue selectivity and tumor targeting ability is a common problem of CPPs, and its clinical application in cancer treatment is greatly limited. Besides co-modified with other targeted ligands, multifunctional CPPs are also under study. As shown in the Fig. 6, CB5005 is constituted by a unique membrane-permeable sequence (CB5005M) cascading to a NF-κB nuclear localization sequence (CB5005N). Zhang et al. confirmed that CB5005 could be effectively taken up by brain capillary endothelial cell bEnd.3 and glioma cells U87 not only by penetrating the cells but also entering into their nuclei [156]. In three-dimensional glioma spheroids, CB5005 was capable of permeating deeply into the tumor spheroids of U87 cell as well. In vivo imaging illustrated that the CB5005 could distribute in the brain and accumulate at the tumor site after intravenous injection. In further research, Zhang et al. loaded doxorubicin with CB5005-coupled liposomes and investigated its potential application in treatment of glioma [156, 157]. The in vitro cytotoxicity exhibited that drug loaded CB5005 liposomes had a synergistic inhibiting effect on U87 cells. Moreover, CB5005 inhibited the growth of tumor when applied alone, and displayed a synergistic anti-tumor effect with DOX in a nude mice xenograft model. CB5005-modified liposomes not only have the unique ability to transfer drugs to tumor sites, but also have synergistic effect on the chemotherapy of glioma and other human tumors. It is a promising tumor drug delivery system. Other CPPs modified vehicles for delivery drug, gene, and macro-molecule for potential application of brain tumor therapy were summarized in Table3.

**Table 3:** Cell-Penetrating Peptides modified nanoparticles for CNS diseases

CPPs-nanoparticles	CPPs	CPPs Sequence	Ligand	Nanoparticles	Cargos	Cell model	Animal model	Therapeutic use	Refs.
TGN-NP	TGN	TGNYKALHPHNG	–	PEG-PLGA nanoparticles	–	bEnd.3	Nude mice, ICR mice	Glioma	[182]
AsTNP	TGN	TGNYKALHPHNG	AS1411	PEG-PCL nanoparticles	Docetaxel	C6, bEnd.3	C6 bearing mice	Glioma	[141]
Tf-PFV-Lip	PFV	PFVYLI	Tf	Liposomes	Doxorubicin, Erlotinib	In vitro BBB model, 3D tumor model	–	Glioma	[183]
ILNPs	IL-13p	TAMRAVDKLLLHLKKLFREGQFNRNFESIIICRDRT	–	Liposomes	Docetaxel	U87, HUVEC, 3D tumor model	U87 bearing mice	Glioma	[140]
cFd-Lip	dNP2	KIKKVKKKGRKKIKKVKKKGRK-cys	Folic acid (FA)	Liposomes	Paclitaxel	4T1, NIH 3T3, bEnd.3, 3D tumor model	Breast tumor bearing mice, breast cancer brain metastasis models	Breast cancer and brain metastasis	[184]
cFd-Lip	dNP2	KIKKVKKKGRKKIKKVKKKGRK-cys	FA	Liposomes	Paclitaxel	bEnd.3, C6	C6 bearing mice	Glioma	[185]
MT1-NP	MT1-AF7p	HWKHLHNTKTFLC	iRGD	PEG-PLA nanoparticles	Paclitaxel	C6	C6 bearing mice	Glioma	[186]
CNP, ANP	CPP, ACPP	CRRRRRRRRR, EEEEEEEEC6GSGRSAGRRRRRRRRC6C	–	Nanoparticles	Paclitaxel	C6	C6 bearing mice	Glioma	[187]
DGL-PEG-LNP	NoLS	KKRTLRKNDRKKRC	–	Nanoparticles	Plasmid DNA encoding inhibitor of growth 4 (ING4)	BCEC	U87 bearing mice	Glioma	[33]
RGE-LS	RGERPPR	RGERPPR	–	Liposomes	Doxorubicin	U87	U87 bearing mice,	Glioma	[188]
TBCNT@OXA	TAT	YGRKKRRQRRR	–	Carbon nanotube	Oxaliplatin	HBMEC, C6	C6 bearing mice	Glioma	[189]
TR-Lip	TR	c(RGDfK)- AGYLLGHINLHHLAHL(Aib)HHIL-Cys	–	Liposomes	Paclitaxel	C6, bEnd.3, CSCs	C6 bearing mice	Glioma	[190]
RGE-PEG / PGG-PTX NPs	RGERPPR	RGERPPR	–	Nanoparticles	polymer-drug conjugates (PGG-PTX)	U87, HUVECs	U87 bearing mice	Glioma	[191]
pSiNPs	SIWV	SIWV	–	Nanoparticles	SN-38	U87	U87 bearing mice	Glioma	[192]
Tf-Pen lip	Pen	RQIKIWFQNRRMKWKK	Tf	Liposomes	Doxorubicin, Erlotinib	U87, bEnd.3	U87 bearing mice	Glioma	[193]
Tf-Pen lip	Pen	RQIKIWFQNRRMKWKK	Tf	Liposomes	5-Fluorouracil	U87, bEnd.3	U87 bearing mice	Glioma	[194]
iNGR-SSL	iNGR	CC(Acm)RNGRGPDC(Acm)	–	Liposomes	Doxorubicin	U87, HUVECs	U87 bearing mice	Glioma	[195]
P1NS/TNC-FeLP	P1NS	Cys-GRKKRRQRRRPQ	TN-C	Thermosensitive liposomal	Doxorubicin	bEnd.3, CRL-2299, C8-D1A, CRL-2541, U87, HTB-14	-	Glioma	[196]
TF-CPP-SSL	CPP	GGRRRRRRRRR-amide	Tf	Liposome	Doxorubicin	C6, HUVECs	C6 bearing mice	Glioma	[197]

## Conclusion and perspectives

Overall, the CNS is highly protected from the influence of exogenous substances and endogenous substances pass through many barrier structures, of which BBB is the most critical one. This makes it very difficult to effectively deliver drugs to the brain lesions. One of the most urgent problems to be solved is to find an effective, reliable and non-toxic method to cross or bypass the BBB. For this reason, minimally invasive or non-invasive methods should be explored to a greater extent. With the development of nanotechnology, it is a good strategy to use nanomaterials to treat CNS disease through intravenous administration and trans-nasal administration. But what cannot be ignored is the neurotoxicity of nanomaterials, which is related to the size, shape, and surface modification of nanoparticles. Nanoparticles with good biocompatibility should be selected for treatment and the dosage of nanomaterials should be strictly controlled.

CPPs can be used as drugs, vectors and ligands in the delivery system to treat CNS diseases. The combination of CPPs and nanomaterials has a good development prospect in the treatment of CNS diseases. However, there are still some limitations that need to be addressed to promote its use. The physicochemical properties changes and pharmacokinetic issues of nanoparticles after CPPs modification should be noted, and the density of CPPs on nanoparticles should also be studied. To achieve safer and more effective nano formulations.

## Abbreviations

CNS: Central nervous system
CPPs: Cell-penetrating peptides
BBB: Blood–brain barrier
P-gp: P-glycoprotein
RES: Reticuloendothelial system
AD: Alzheimer’s disease
PD: Parkinson’s disease
GBM: Glioblastoma multiforme
RTK: Receptor tyrosine kinase
P13K: Phosphatidylinositol-3-OH kinase
MARCKS: Myristoylated alanine-rich C-kinase substrate
GIC: Glioma initiating cells
Cx43: Connexin43
ANXA1: Annexin A1
NTS: Nuclear translocation signal
PG-1: Peptide protegrin-1
ROS: Reactive oxygen species
hMT1A: Human metallothionein 1a
RAS: Rasagiline
LRP1: Lipoprotein receptor-related protein-1
miRNA: MicroRNA
MMP: Matrix metalloproteinase
Tf: Transferrin
HIV: Human immunodeficiency virus
RMT: Receptor mediated cell transport
PAMAM: Polyamideamide
BMPs: Bacterial magnetic nanoparticles
PEG-PCL: Polyethylene glycol-polycaprolactone
